# A Case of Inverted Colonic Diverticulum

**DOI:** 10.7759/cureus.59460

**Published:** 2024-05-01

**Authors:** Maysa Al-Badawi, Omar G Alturkmani, Shafeq G Al Turkmani, Shahd A Attar, M. Haitham Al-Midani

**Affiliations:** 1 General Medicine, Digestive Disease and Nutrition Center, Burton, USA; 2 College of Medicine, Hama University, Hama, SYR; 3 Gastroenterology, Digestive Disease and Nutrition Center, Burton, USA

**Keywords:** diagnosis of inverted colonic diverticulum, colonoscopy complications, perforation of colon, colonic polyps, inverted colonic diverticulum

## Abstract

Inverted colonic diverticulum (ICD) is an infrequent finding on colonoscopy, often misdiagnosed as colonic polyps. Further endoscopic intervention, such as polypectomy or biopsy, may lead to colonic perforation. For that reason, the endoscopist should be aware of the possibility of detecting these lesions when performing a colonoscopy. Diagnosing an ICD can be confirmed by inspection and gentle eversion using the probe. In this case report, we present a patient who was found to have inverted colonic diverticulum as we highlight the importance of distinguishing it from colonic polyps in order to prevent severe complications.

## Introduction

Colonic diverticular disease (DD) is a very common finding on colonoscopies seen as outpouchings of the intestinal wall occurring in 5% of cases at 40 years and reaching up to 65% at age 80 [1]. Inverted colonic diverticulum (ICD) is a rare finding, found in 0.7-1.7% of cases, with a mean age of 62 years old. There is a slight increase of prevalence in males in comparison to females [1,2]. It is most commonly seen in the sigmoid colon [1,2]. Inverted diverticulum (ID) usually presents without a stalk which makes it indistinguishable from polyps [1]. If an attempt for endoscopic resection is done, it may end in colonic perforation [1]. ID endoscopic features and some colonoscopic maneuvers help in distinguishing ICD from colonic polyps. In this article, we present a case of inverted colonic diverticulum.

## Case presentation

**Figure 1:**
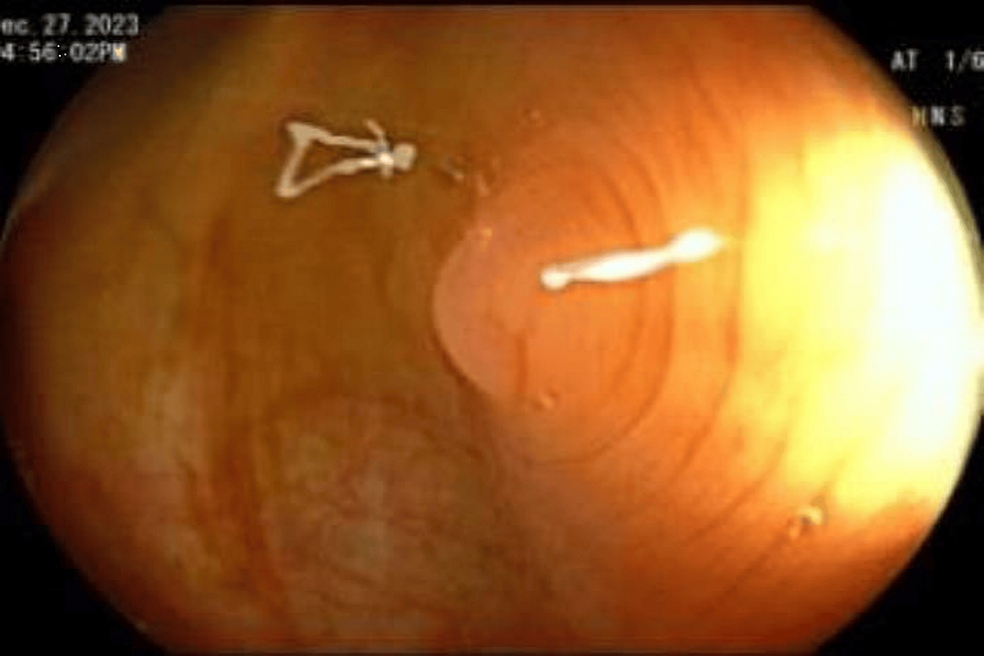
Inverted diverticulum at the sigmoid colon Shiny mucosal lesion, similar to the surrounding mucosa with concentric rings around it.

**Figure 2:**
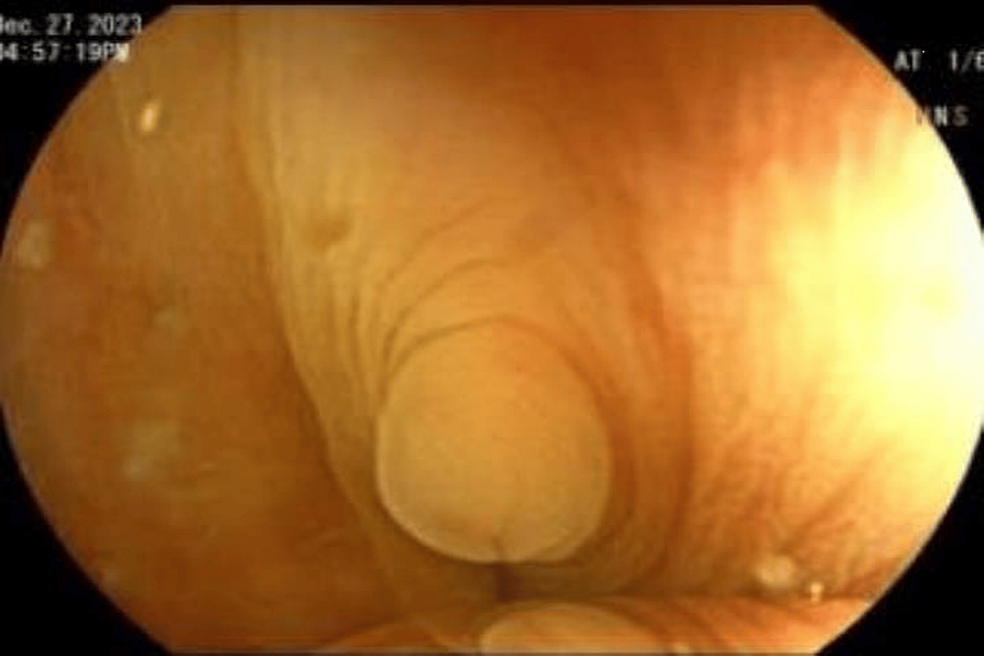
Another view of the inverted colonic diverticulum at the sigmoid colon Lesion surrounded by concentric rings, resembling a polyp with no stalk.

**Figure 3:**
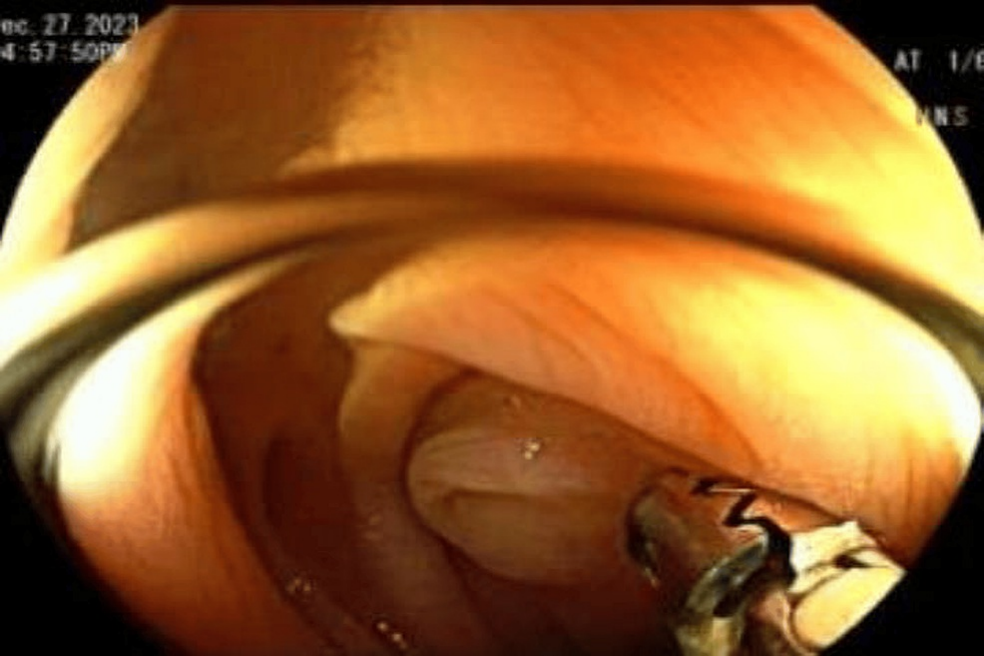
Probing at the sigmoid colon Probing with cold forceps to look for invagination of the lesion.

**Figure 4:**
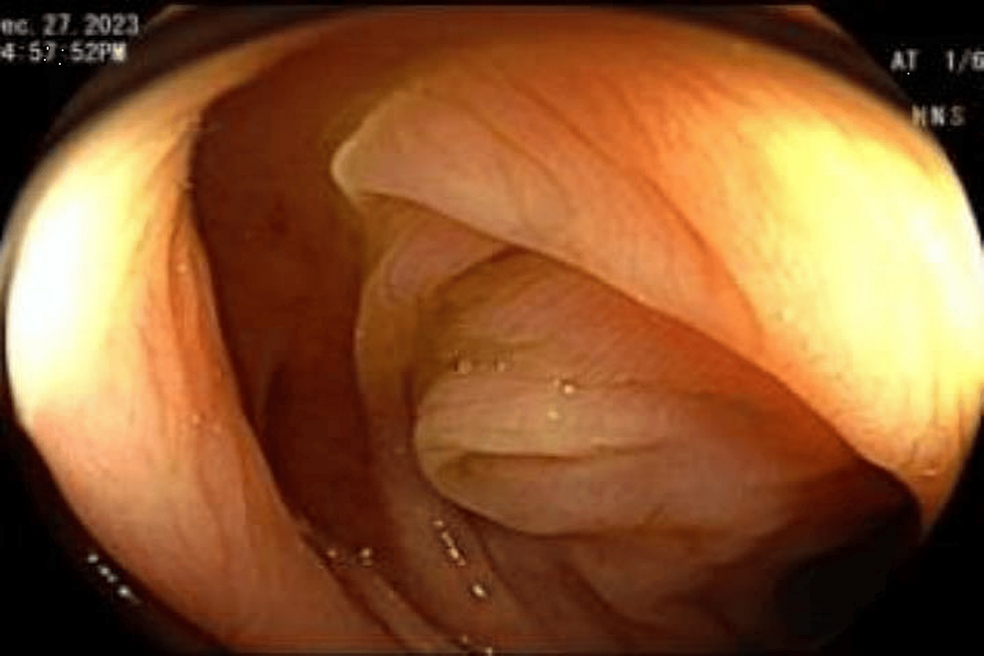
Sigmoid colon post probing Eversion of inverted diverticulum post probing.

**Figure 5:**
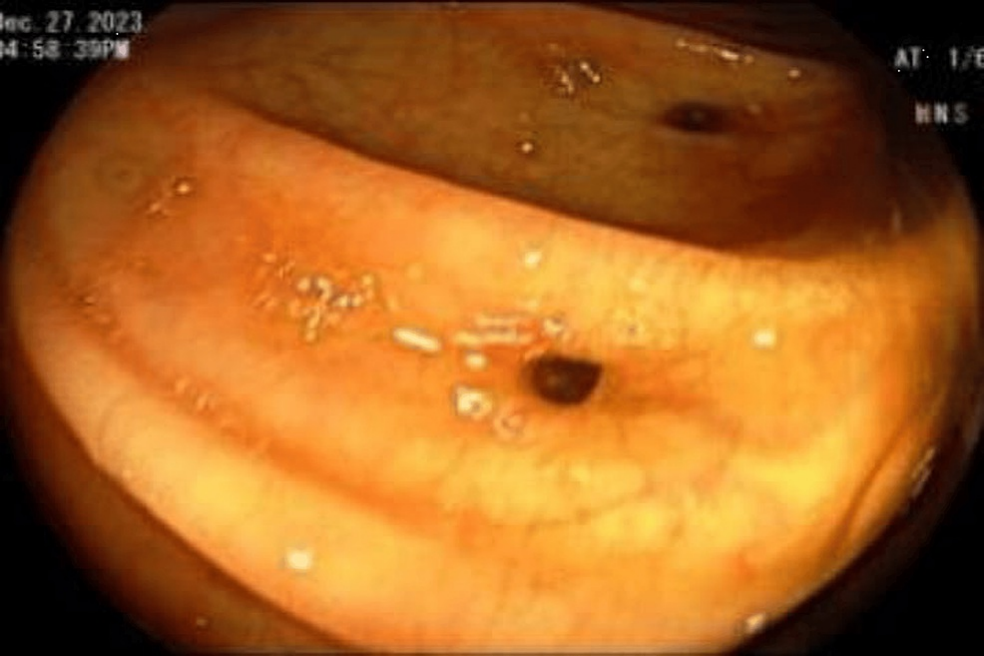
Descending Colon Diverticulosis

**Figure 6:**
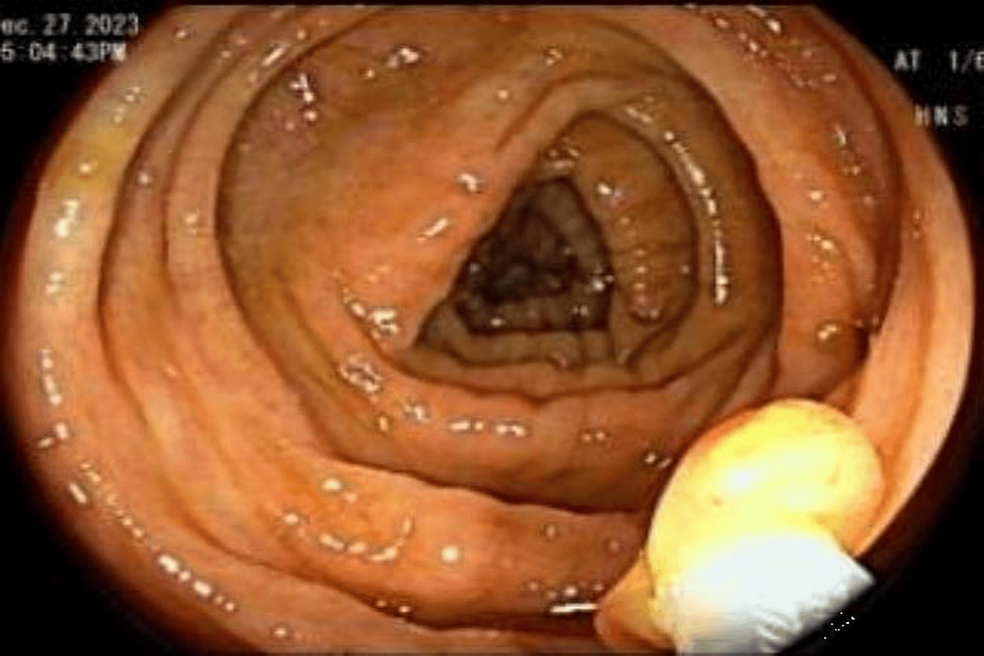
Descending Colon Polyp 7mm polyp at 60cm at the descending colon.

A 56-year-old male, with past medical history of hyperlipidemia, was referred for colonoscopy screening for history of colonic polyps seen on a previous colonoscopy examination which was done eight to 10 years ago per patient. Patient had no gastrointestinal complaints besides some occasional heartburn relieved by over-the-counter Tums as needed. Colonoscopy examination with artificial intelligence (AI) technology revealed non-specific mucosal inflammation in the sigmoid colon for which a cold forcep biopsy was taken, which revealed colonic mucosa with focal surface sloughing and mildly thick basement membrane with early collagenous colitis not excluded. A shiny mucosal lesion, similar to the surrounding mucosa resembling a polyp with concentric rings around it and no stalk, was seen at the sigmoid colon as well (Figure 1, Figure 2). Using the cold forceps biopsy, probing lead to invagination of the lesion (Figure 3, Figure 4). The diagnosis of inverted colonic diverticulum was made. Diverticulosis was seen in the descending colon (Figure 5). In addition, polypectomy of a 7mm polyp at 60cm at the descending colon was done along with mono-polar ablation via tip of snare to eliminate all residual polyp tissue near polypectomy site and to control bleeding (Figure 6). Polyp pathology showed focal crypt serrated hyperplasia with differential of early serrated polyp vs. hyperplastic polyp. The patient tolerated the procedure well and there were no complications.

## Discussion

Colonic DD is a very common finding on colonoscopies seen as outpouchings of the intestinal wall occurring in 5% of cases at 40 years and reaching up to 65% at age 80 [1]. ICD is a rare finding, found in 0.7-1.7% of cases, with a mean age of 62 years old. There is a slight increase of prevalence in males in comparison to females [1,2]. It is most commonly seen in the sigmoid colon [1,2]. According to a study done by Gulaydin et al. 2021, among 810 patients who underwent a colonoscopy between April 2016 and November 2019, ICD was observed in 1.73%, including 11 men and three women and a case of colon perforation was reported after polypectomy with hot biopsy forceps which was treated by surgical operation [2]. For that reason, differential diagnosis of ID is crucial during performing colonoscopy. ID usually presents without a stalk which makes it indistinguishable from polyps [1]. If an attempt for endoscopic resection is done, it may end in colonic perforation [1]. ID endoscopic features include a smooth appearance with pink shiny mucosa which is similar to the surrounding normal mucosa, central indentation is sometimes present, and concentric rings around the ID with normal colonic pit pattern [1]. It can also be differentiated from colonic polyps by colonoscopy maneuvers, which include empty and soft structures on palpation, continual probing using the cold forceps biopsy lead to invagination of the lesion, partial eversion or flattening when using water (water jet deformation sign), and reversal of ID with air insufflation [1]. Recurrent diverticulitis might evoke the development of non-neoplastic polyps within the diverticulum, which complicates the ID endoscopic assessment [1]. A case of ICD was reported in October 2021 by Chang during which the ICD was seen as a small 0.3cm sessile polypoid lesion in the sigmoid solon with several pale concentric folds (aurora rings) surrounding the lesion which were enhanced by narrow-band, also direct water jet reverted the lesion to a typical diverticular appearance [3]. A case of intussusception caused by a large ICD near the ileo-cecal valve was reported by Zhang et al. (2018) [4]. The patient underwent laparoscopic right hemicolectomy and histopathological results showed that ICD diameter was 3.8cm which was seen on the side of the ascending colon, resulting in ileocolonic intussusception and intestinal necrosis [4]. 

## Conclusions

In conclusion, it is important to distinguish ICD from colon polyps by endoscopic features and colonoscopic maneuvers in order to prevent colonic perforation with any attempt of resection mandating an emergency surgery. 
